# Sirtuin 1 in Endothelial Dysfunction and Cardiovascular Aging

**DOI:** 10.3389/fphys.2021.733696

**Published:** 2021-10-06

**Authors:** Stefano Ministrini, Yustina M. Puspitasari, Georgia Beer, Luca Liberale, Fabrizio Montecucco, Giovanni G. Camici

**Affiliations:** ^1^Center for Molecular Cardiology, University of Zurich, Zurich, Switzerland; ^2^Internal Medicine, Angiology and Atherosclerosis, Department of Medicine and Surgery, University of Perugia, Perugia, Italy; ^3^First Clinic of Internal Medicine, Department of Internal Medicine, University of Genoa, Genoa, Italy; ^4^Istituto di Ricerca e Cura a Carattere Scientifico Ospedale Policlinico San Martino Genoa–Italian Cardiovascular Network, Genoa, Italy; ^5^Department of Cardiology, University Heart Center, University Hospital Zurich, Zurich, Switzerland; ^6^Department of Research and Education, University Hospital Zurich, Zurich, Switzerland

**Keywords:** sirtuin (SIRT1), aging–old age–seniors, eNOS (endothelial nitric oxide synthase), inflammaging, endothelial (dys)function, atherosclerosis, cardiovascular disease

## Abstract

Sirtuin 1 (SIRT1) is a histone deacetylase belonging to the family of Sirtuins, a class of nicotinamide adenine dinucleotide (NAD+)-dependent enzymes with multiple metabolic functions. SIRT1 localizes in the nucleus and cytoplasm, and is implicated in the regulation of cell survival in response to several stimuli, including metabolic ones. The expression of SIRT1 is associated with lifespan and is reduced with aging both in animal models and in humans, where the lack of SIRT1 is regarded as a potential mediator of age-related cardiovascular diseases. In this review, we will summarize the extensive evidence linking SIRT1 functional and quantitative defects to cellular senescence and aging, with particular regard to their role in determining endothelial dysfunction and consequent cardiovascular diseases. Ultimately, we outline the translational perspectives for this topic, in order to highlight the missing evidence and the future research steps.

## Introduction

Aging is defined as the result of a progressive functional decay in multiple tissues and physiologic functions. Cellular senescence, defined as a permanent arrest of the cell cycle, and the subsequent progression toward apoptosis, is one of the cellular mechanisms contributing to the loss of tissue regenerative potential and progressive loss of function with aging (Hernandez-Segura et al., 2018).

Overall, aging is associated with an increased risk of diseases and death, with different organs and systems being differently influenced by aging, and whereby the cardiovascular system is among the most severely hit. As a result, age represents the most relevant risk factor for cardiovascular and cerebrovascular diseases (Camici et al., 2015). Accordingly, the possibility to address the molecular mechanisms underlying aging of the cardiovascular system is an exciting perspective in order to reduce the burden of death and disability associated with cardiovascular diseases.

The interest in Sirtuins emerged as pioneering studies demonstrated an increased lifespan of yeast Saccharomyces cerevisiae with Sir2 (Silent information regulator 2) overexpression, whereas a loss of function of Sir2 leads to defect in epigenetic silencing, DNA repair process, and shorter lifespan (Kaeberlein et al., 1999). Such effect was then demonstrated in animals, Caenorhabditis elegans and Drosophila, showing the highly conserved roles and domains of Sir2 (Tissenbaum and Guarente, 2001; Rogina and Helfand, 2004; Viswanathan et al., 2005). Mammals express seven homologs of Sir2, named Sirtuins (i.e., SIRT1–SIRT7) (Yamamoto et al., 2007). Sirtuins are located in different cellular compartments and exert their role by acting on different targets, in a non-redundant manner (Liberale et al., 2020a; Puspitasari et al., 2021). To date, Sirtuin 1 (SIRT1), localized in the nucleus, is the most well-studied and the best characterized of mammalian sirtuins.

This narrative review is based on the material available on PubMed as of June 2021. The following search terms were employed: “Sirtuins; SIRT1” in combination with “endothelial (dys)function; cardiovascular disease.”

In the following paragraphs we will summarize the existing evidence about the pathophysiological role of SIRT1 in atherosclerotic cardiovascular disease (ASCVD), and in particular in one of its main functional features, namely endothelial dysfunction (ED).

## A Paradigm for Cardiovascular Aging: the Role of Sirtuins

Sirtuins are a family of nicotinamide adenine dinucleotide (NAD+)-dependent enzymes that catalyze histone and non-histone deacetylation of lysine residues (Guarente, 2011; Camici et al., 2015; Winnik et al., 2015). Sirtuins belong to class III histone deacetylases, and their catalytic activity is regulated by the dynamic changes of NAD+ level and NAD+/NADH ratio (Grabowska et al., 2017). Due to their dependency on NAD+ as co-substrate, sirtuins have been implicated in various cellular processes, including modulation of cellular redox state and mediation of heterochromatin formation (Dang, 2014; Singh et al., 2018). Multiple studies demonstrated the involvement of sirtuins in glucose and lipid metabolism, suggesting their role in maintaining metabolic health (Houtkooper et al., 2012).

SIRT1 is the closest mammalian homolog of the yeast Sir2 protein (Michan and Sinclair, 2007). SIRT1 was demonstrated to participate in various biological processes, including DNA repair, inflammation, autophagy, and longevity. Thus, its function was associated to several diseases, including ASCVD (Haigis and Guarente, 2006; Chen et al., 2020). A large number of proteins were identified as substrates of SIRT1. The first recognized function of SIRT1 is deacetylation of lysine residues of histone protein H1, H3, and H4, thus modulating chromatin structure and expression of target genes (Singh et al., 2018; Chen et al., 2020). Besides, non-histone proteins were recognized as its targets, including p53, nuclear factor kappa-light-chain-enhancer of activated B cells (NF-κB), and the forkhead box O class (FOXO) transcription factors.

To date, the association of SIRT1 with aging and longevity has been described in human and non-human mammalians. Specifically, aging is associated with a significant decrease of SIRT1 activity and expression in several organs and tissue, including the cardiovascular system (Braidy et al., 2011; Donato et al., 2011). This phenomenon is partially driven by the decline in NAD+ levels observed with aging, yet experimental evidence demonstrated that SIRT1 inhibition leads to genomic instability and development of a senescent phenotype in endothelial cells, irrespective of NAD+ bio-availability (Mostoslavsky et al., 2006; Ota et al., 2007).

Senescence of endothelial cells is the result of several mechanisms, such as DNA injury, telomeres shortening below the critical length of 50–200 base pairs, mitochondrial dysfunction with accumulation of reactive oxygen species (ROS), and impaired proteostasis, following a failure of lysosomal protein degradation through autophagy or the ubiquitin-proteasome system (Laina et al., 2018).

Sirtuins have been reported to participate in DNA single-chain and double chain damage repair by deacetylating the DNA repair machinery components (Yamamori et al., 2009; Alves-Fernandes and Jasiulionis, 2019; Lagunas-Rangel, 2019) and p53, a crucial process for cell recovery after DNA injury (Yamamori et al., 2009). Furthermore, SIRT1 promotes the elongation of telomeres (Palacios et al., 2010) through the induction of the telomere reverse transcriptase (TERT), mediated by c-Myc activation (De Bonis et al., 2014).

SIRT1 indirectly modulates mitochondrial ROS production and promotes the expression of antioxidants through the induction of the FOXO transcription factors (Maiese, 2021). Sources of ROS include mitochondrial respiration, catabolism of purine bases through xanthine oxidase, cyclooxygenases, and nicotinamide adenine dinucleotide phosphate (NADPH) oxidase (Panth et al., 2016). These processes are ubiquitous in mammals' tissues and, therefore, the redox balance is maintained in a steady state by endogenous anti-oxidant systems (Wu et al., 2004; Panth et al., 2016; Bacchetti et al., 2020). SIRT1 plays an active role in the cellular defense against oxidative stress and, at the same time, its function is affected by the presence of ROS through post-translational modifications (Hwang et al., 2013).

FOXO transcription factors are also involved in inflammation and autophagy, together with the mechanistic target of rapamycin (mTOR) (Singh et al., 2018; Cheng, 2019; Chen et al., 2020). The latter is a negative regulator of autophagy and impaired autophagy associates to accumulation of oxidative damage, loss of proteostasis, genomic instability and epigenetic alteration, inducing cellular senescence (Rajendran et al., 2019; Stead et al., 2019). Senescent cells, although quiescent under a replicative point of view, are metabolically active and develop a peculiar senescence associated secretory profile (SASP), consisting in molecules with prevalent pro-inflammatory effects (Cayo et al., 2021). Activation of mTOR was associated with both a reduction and an enhancement of autophagy and, consequently, with both a promotion and a prevention of senescence (Laberge et al., 2015; Sung et al., 2018). As proposed by Cayo et al. the effect of mTOR on autophagy probably depends on the cellular senescence status (Cayo et al., 2021). Takeda-Watanabe and coll. found that SIRT1 inhibition results in increased phosphorylation of mTOR and its downstream target, p70-s6 kinase, with a net decrease in autophagy and increase in inflammation in macrophages (Takeda-Watanabe et al., 2012).

Inflammation has a relevant impact on endothelial dysfunction and aging; this notion was recently reinforced with the coining of the new term “inflamm-aging” (Liberale et al., 2020b). In this context, oxidative stress plays a pivotal role in the development of inflamm-aging through the activation of different intracellular pathways converging on the transcription factor NF-κB (Morgan and Liu, 2011) and leading to the release of pro-inflammatory cytokines and pro-thrombotic factors. SIRT1 interacts with NF-κB through the RelA/p65 subunit and deacetylates it at Lys310, leading to inhibition of signaling and suppression of inflammation (Yeung et al., 2004). Conversely, NF-κB down-regulates SIRT1 activity through the expression of miR-34a, interferon γ (IFNγ), and ROS (Kauppinen et al., 2013).

Endothelial dysfunction (ED) is a main feature of cardiovascular aging. It is defined as the failure of endothelium to mediate an adequate vasodilatatory response to hypoxia or hemodynamic stimuli, such as shear stress (Lüscher and Corti, 2004; Godo and Shimokawa, 2017). ED is associated with a pro-inflammatory and pro-thrombotic status, and eventually with an increased risk of cardiovascular events (Bonetti et al., 2004; Kitta et al., 2009).

Endothelial cells exert their functions through multiple, redundant, molecular pathways, the most relevant being nitric oxide (NO) production. ED is characterized by a reduced NO bioavailability, to such an extent that this itself is also considered as a definition for ED (Dimitris et al., 2011). NO is a soluble radical with vasodilatatory, anti-inflammatory, anti-adhesive and anti-thrombotic properties, produced by the oxidation of L-arginine to L-citrulline, catalyzed by the enzyme nitric oxide synthase (NOS) (Cyr et al., 2020). Endothelial cells are characterized by a constitutively expressed isoform of NOS, named endothelial NOS (eNOS), whereas many other cell types can express the inducible isoform of NOS (iNOS) under cytokines stimulation (Förstermann and Sessa, 2012).

The imbalance between substrate availability and NO production by eNOS is termed eNOS uncoupling and is a pivotal mechanism of endothelial dysfunction. The main cause of eNOS uncoupling is the unbalance of the redox state toward oxidation, caused by ROS (Yoshida and Kisugi, 2010; Bacchetti et al., 2020). Other causes of eNOS uncoupling, include asymmetric dimethyl-L-arginine (ADMA) (Susanne et al., 2014) and enzymatic post-translational modifications of eNOS (Fleming and Busse, 2003; Chen et al., 2010). Finally, the bioavailability of L-arginine itself can be reduced by the activity of arginases, produced by endothelial and inflammatory cells (Berkowitz et al., 2003).

SIRT1 promotes NO availability through different direct and indirect mechanisms. Most importantly, SIRT1 has a direct activating effect on eNOS through deacetylation at Lys496 and Lys506 (Mattagajasingh et al., 2007). Interestingly, a positive feedback between NO and SIRT 1 exists, since NO promotes in turn the transcription of SIRT1 (Arunachalam et al., 2010; Caito et al., 2010). Additionally, SIRT1 inhibits the transcription of the adaptor protein p66^Shc^ through deacetylation of the histone protein H3 in the promoter region. Since p66^Shc^ promotes the mitochondrial formation of ROS and inhibits the transcription of the antioxidant enzyme superoxide dismutase 2 (SOD2), its downregulation results in a net antioxidant effect (Di Lisa et al., 2009; Trinei et al., 2013). Finally, SIRT1 was reported to induce the nuclear factor erythroid 2—related factor 2 (Nrf2), a transcription factor having a key role in the expression of the most relevant anti-oxidant enzymes, such as glutathione S-transferase Ya (GST Ya) subunit, heme oxygenase 1 (HO-1), and γ-glutamylcysteine synthetase (γ-GCS) (Kaspar et al., 2009; Kawai et al., 2011; Ma et al., 2019).

**Figure 1:**
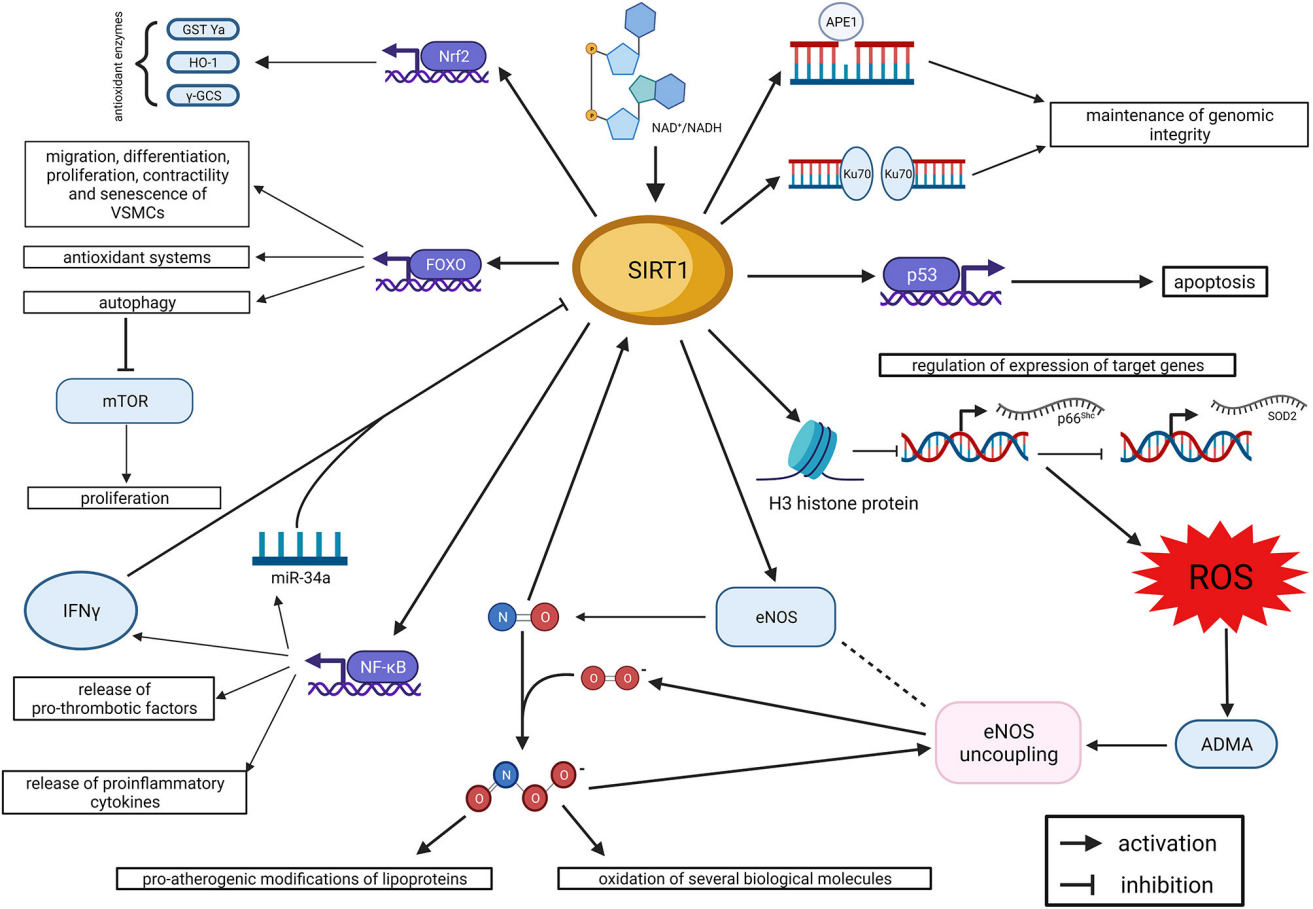
SIRT1 and endothelial dysfunction. Summary of molecular mechanisms (created with BioRender.com). ADMA, asymmetric dimethyl-arginine; APE1, apurinic/apyrimidinic endonuclease 1; eNOS, endothelial nitric oxide synthase; FOXO, forkhead box O class; γ-GCS, γ-glutamylcysteine synthetase; GST Ya, glutathione S-transferase Ya; HO 1, heme oxygenase 1; IFNγ, interferon γ; mTOR, mechanistic target of Rapamycin; NAD: nicotinamide adenine dinucleotide; NF-κB, nuclear factor kappa-light-chain-enhancer of activated B cells; Nrf2, nuclear factor erythroid 2—related factor 2; ROS, reactive oxygen species; SIRT1, Sirtuin 1; SOD2, superoxide dismutase 2.

By regulating the balance between oxidant and anti-oxidant systems, SIRT1 holds specific functions at the level of the endothelium and arterial wall. The main physiologic effects of SIRT1 in the endothelium are summarized in Figure 1. More in general, SIRT1 preserves endothelial function and integrity from the detrimental effects of oxidative stress. Indeed, oxidative stress induces premature cell senescence and apoptosis in endothelial cells through the acetylation of FOXO3 and p53, whereas SIRT1 promotes their deacetylation and, eventually, cell survival (Brunet et al., 1999, 2004; Ota et al., 2007, 2008). Noteworthy, the acetylation of FOXO3, induced by oxidative stress, is able to promote also ROS detoxification and DNA repair (Kops et al., 2002; Tran et al., 2002); so, it is expected to have a dual effect on endothelial cells survival. Interestingly, the deacetylating effect of SIRT1 on FOXO3 does not affect its ability to arrest the cell cycle and provide ROS detoxification and DNA repair (Brunet et al., 2004). Furthermore, FOXO3 regulates multiple functions of vascular smooth muscle cells (VSMCs), including migration, differentiation, proliferation, contractility and senescence (Allard et al., 2008; Huang et al., 2015; Jin et al., 2015; Wang et al., 2015; Liu et al., 2018). In particular, the SIRT1/FOXO3 axis has been demonstrated to promote differentiation and contractility of VSMCs (Huang et al., 2015; Liu et al., 2018), and these novel mechanisms could contribute to the overall effect of SIRT1 on vascular function.

Finally, oxidative stress promotes endothelial-to-mesenchymal transition, a pivotal process in neoangiogenesis and vascular development, but associated with cardiovascular diseases and ED, too (Kovacic et al., 2019).

Chronic low-grade systemic inflammation was identified as a main feature at the crossroad of aging, atherosclerosis and ED (Steyers and Miller, 2014; Ministrini et al., 2021). Pro-inflammatory cytokines, such as tumor necrosis factor α (TNF-α), interleukin 1 (IL-1) and IFN- γ, have a detrimental effect on endothelial function, promoting the apoptosis of endothelial cells and impairing the bioavailability of NO through multiple mechanisms, such as inhibition of eNOS gene expression, degradation of eNOS mRNA, inhibition of ADMA degradation and promotion of ROS production by NADPH oxidase (Carbone and Montecucco, 2015). As reported above, SIRT1 interferes with the signaling of these pro-inflammatory molecules, through inhibition of NF-κB and promotion of the immune-modulatory FOXO transcription factors (Peng, 2008), thus exerting an overall anti-inflammatory effect and blunting the age-related ED.

## SIRT1 in Cardiovascular Diseases

As a ubiquitous molecule with a role in multiple cell functions, the relationship between SIRT1 and cardiovascular disease is not limited to endothelial function. In particular, SIRT1 counteracts the progression of atherosclerotic lesions through different actions (Zhang et al., 2008), including oxidation of lipoproteins (Stein et al., 2010), sub-endothelial inflammatory cells infiltration (Breitenstein et al., 2013), senescence of endothelial progenitor cells (Ming et al., 2016), neo-intima proliferation (Li et al., 2011) and plaque destabilization (Xia et al., 2012).

Furthermore, SIRT1 has a glucose lowering action, since it promotes insulin secretion and the peripheral utilization of glucose (Strycharz et al., 2018). As it is well-known, hyperglycemia, insulin resistance and diabetes mellitus are major determinants of cardiovascular diseases, and the glucose-lowering property of SIRT1 is thought to contribute to its protective function toward cardiovascular diseases.

Moreover, SIRT1 holds important roles also in cardiomyocytes, where it exerts anti-apoptotic functions (Matsushima and Sadoshima, 2015), counteracts endoplasmic reticulum stress (Prola et al., 2017), increases myocardial contractility (Hsu et al., 2017) and resistance to ischemia/reperfusion injury (Wang et al., 2018). SIRT1 is upregulated during pressure overload, caloric restriction and physical exercise, whereas it is downregulated during acute ischemia (Matsushima and Sadoshima, 2015; Najafipour et al., 2021). Interestingly, also constitutional overexpression of SIRT1 in transgenic mice was associated with a reduced contractile function of the myocardium (Alcendor et al., 2007; Kawashima et al., 2011) and, therefore, a U-shaped dose-response curve was hypothesized for the relationship between SIRT1 and myocardial function.

Considering the above, SIRT1 is expected to have a relevant effect on the burden of ASCVD in humans. Consistently, a reduced intracellular expression of SIRT1 gene was observed in patients with stable coronary artery disease (sCAD) and acute coronary syndrome (Breitenstein et al., 2013; Hu et al., 2015), whereas increased circulating levels were associated with sCAD (Kilic et al., 2014). Conversely, lower levels of circulating SIRT1 were associated with a history of atrial fibrillation (Kalstad et al., 2021), whereas an increased levels were observed in the left atrial appendage of patients with valvular atrial fibrillation (Sun et al., 2012).

These apparent discrepancies can be explained considering the multiple epigenetic factors differently regulating the expression of SIRT in the various cell types.

More robust evidence for the association between SIRT1 and the ASCVD in humans was collected in genomic studies. Multiple single nucleotide polymorphisms (SNPs) of the SIRT1 gene have been found to associate with ASCVD: the polymorphisms *rs7069102 C*>*G*, localized in the intron 4, and *rs2273773 C*>*T*, localized in the exon 5, are associated with an increased risk of sCAD, an increased level of circulating SIRT1 and a reduced expression of eNOS (Kilic et al., 2014). The polymorphism *rs12413112 A*>*G*, localized in the 3′ untranscribed region, was negatively associated with the presence of CAD (Nasiri et al., 2018), but positively with the presence of carotid intima-media thickness (cIMT) (Kedenko et al., 2014). Conversely, the intronic polymorphism *rs1467568 A*>*G* has been associated with a reduced cIMT (Kedenko et al., 2014), but only in the male sex, and with a borderline significant reduction for the risk of CAD (Nasiri et al., 2018). Ultimately, the polymorphism *rs3758391 T*>*C* located in the gene promoter is associated with a higher expression of SIRT1 mRNA during acute coronary syndromes (Hu et al., 2015).

## Translational Relevance of Sirt1: Where Do We Stand So FAR?

**Figure 2:**
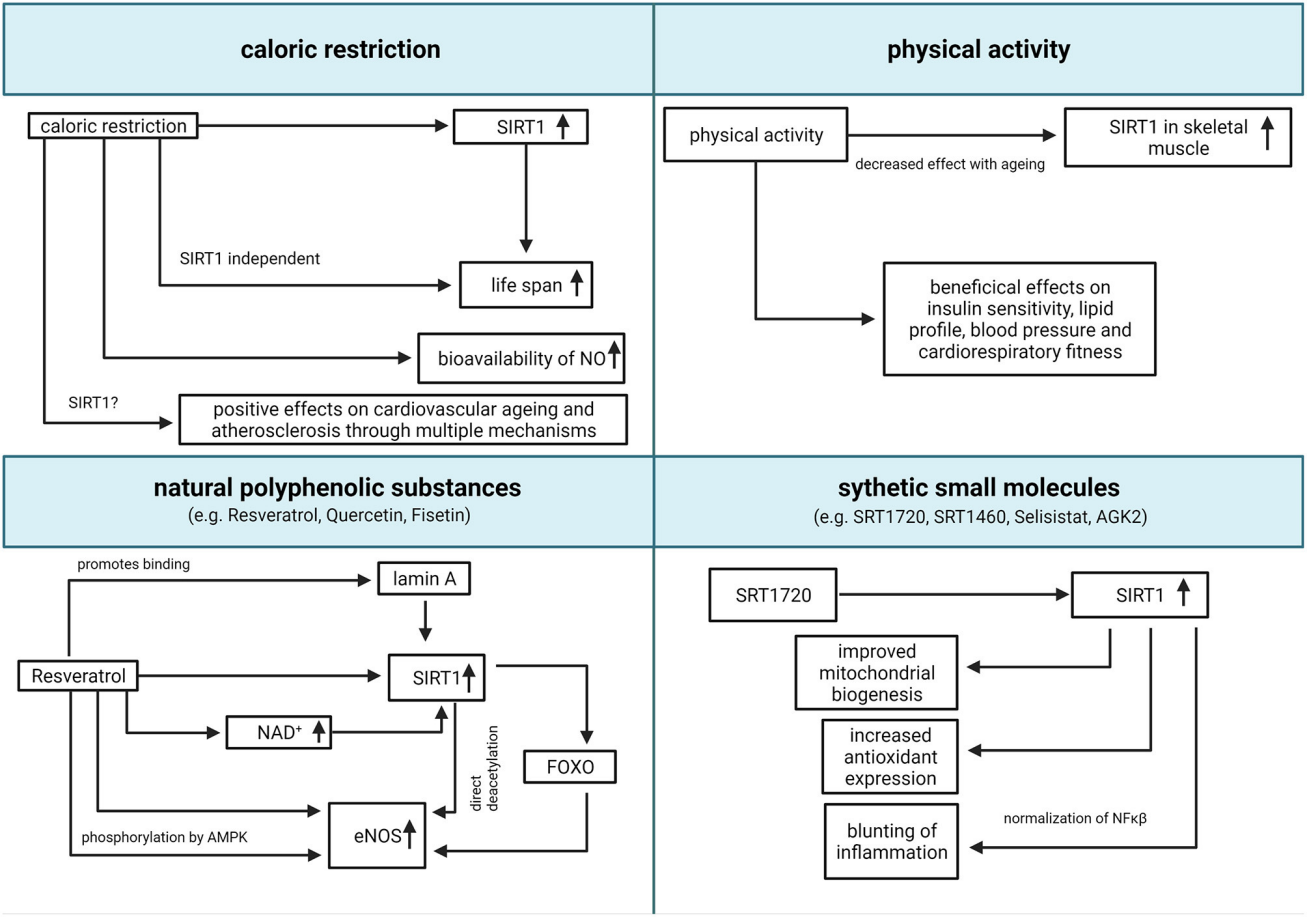
Translational relevance of SIRT1. Summary of potential pharmacologic and non-pharmacologic interventions to increase SIRT1 activity (created with BioRender.com). eNOS, endothelial nitric oxide synthase; FOXO, forkhead box O class; NAD, nicotinamide adenine dinucleotide; NO, nitric oxide; SIRT1, Sirtuin 1; NF-κB, nuclear factor kappa-light-chain-enhancer of activated B cells.

Considering the above-mentioned evidence, the possibility to enhance the activity of SIRT1 through pharmacologic and non-pharmacologic treatments for the management or prevention of age-associated diseases, has become increasingly appealing. The main therapeutic strategies, and the relative claimed mechanisms of action, are summarized in Figure 2.

Caloric restriction (CR) is the most consistent non-pharmacologic intervention to increase lifespan in animal models (Cantó and Auwerx, 2009). It is defined as a moderate reduction of caloric intake (usually 20–49%), compared to a normal *ad libitum* diet, without compromising the intake of essential macro- and micronutrients (Piper and Bartke, 2008). CR was associated with an increased expression of SIRT1 both in animal models and humans (Guarente, 2013), and SIRT1 was suggested as the main mediator of prolonged lifespan after CR since the genetic deletion of SIRT1 reduces the benefits of CR in murine models (Chen et al., 2005). Reciprocally, both high fat diet and obesity lead to a reduced expression of SIRT1 in both animals and humans (Guarente, 2013). Multiple hypotheses were proposed to explain the link between CR, SIRT1, and prolonged lifespan, all involving the bioavailability of NAD+ as limiting factor for the activity of SIRT1 (Cantó and Auwerx, 2009), but none of these hypotheses found an experimental confirmation. Currently, it is widespread opinion that CR leads to lifespan prolongation through multiple biochemical pathways, both Sirtuins-dependent and -independent (Lee et al., 2019).

CR activates eNOS and increases the bioavailability of NO, leading to an improvement of endothelial function in animals and humans (Zanetti et al., 2010; Shinmura, 2011). Overall, CR showed a positive effect on cardiovascular aging and atherosclerosis, both in animals and humans, through multiple mechanisms, including the amelioration of systemic inflammation, ROS production, dyslipidemia, hypertension, and insulin resistance (Abiri and Vafa, 2019). However, to date we are unable to discriminate whether these effects are mediated by SIRT1 and to which extent. Interestingly, it was suggested that some nutritional treatments like intermittent fasting could mimic the effect of CR in humans producing an analog effect on SIRT1 activity (Abiri and Vafa, 2019).

Physical activity (PA) has an established role in cardiovascular prevention, because of its beneficial effects on insulin sensitivity, lipid profile, blood pressure, and cardiorespiratory fitness (Myers et al., 2019). PA was associated with an increased expression of SIRT1 and a reduction of ROS in the skeletal muscle, both in animals and in humans (Pacifici et al., 2019). This effect was observed also in older animals, suggesting that PA could at least partially revert the age-related decay of SIRT1 activity (Ferrara et al., 2008). However, the extent of SIRT1 activation seems to be age-dependent, with a reducing enhancement with advancing age (Huang et al., 2016). Interestingly, PA-induced increased SIRT1 was also observed in animal models of myocardial infarction, and was associated with a reduction of apoptotic markers and overall myocardial damage (Donniacuo et al., 2019), suggesting that SIRT1 could play a role in mediating the beneficial effects of PA after ischemic myocardial damage.

Pharmacologic modulation of SIRT1 activity is also a promising opportunity. Two main classes of substances were observed to enhance SIRT1 activity: natural polyphenolic substances, such as Resveratrol, Fisetin and Quercetin, and synthetic small molecules, such as SRT1720, SRT1460, Selisistat (EX 527), SCIC2.1 and AGK2 (Bai et al., 2018; Scisciola et al., 2020).

Resveratrol has recently gained attention because of its anti-oxidant properties, and several potential health benefits of resveratrol were accordingly proposed. Resveratrol is a natural-derived flavonoid, with several direct and indirect molecular targets, including SIRT1 (Hori et al., 2013; Liberale et al., 2019; Li et al., 2019). Notably, Resveratrol is a non-specific activator of Sirtuins and some of the observed effects could be mediated also by Sirtuins other than SIRT1 (Sun et al., 2021). Resveratrol directly induces the transcription of SIRT1 (Xia et al., 2017), increases the bioavailability of NAD+ through the inhibition of phosphodiesterases (Park et al., 2012) and promotes the binding of SIRT1 to its nuclear activator lamin A (Liu et al., 2012). Interestingly, a mutation of lamin A gene (LMNA) causes, in humans, Hutchinson-Gilford progeria syndrome, characterized by premature death due to cardiovascular diseases (Ahmed et al., 2018). Resveratrol was demonstrated to increase the expression of eNOS *in vitro*, in a SIRT1/FOXO dependent manner (Xia et al., 2013). Moreover, Resveratrol enhances the activity of eNOS, promoting the phosphorylation at Ser-1177 by adenosine-mononucleotide-activated protein kinase (AMPK) (Xu et al., 2009; Heiss and Dirsch, 2014) and the deacetylation at Lys496 and Lys506 by SIRT1 (Mattagajasingh et al., 2007; Arunachalam et al., 2010). Oral administration of resveratrol was demonstrated to improve endothelial function in different animal models of cardiometabolic diseases, such as systemic hypertension (Dolinsky et al., 2013), diabetes mellitus (Zhang et al., 2009) and dyslipidemia (Zou et al., 2003). However, a large meta-analysis did not find any significant difference between subjects treated with Resveratrol and controls in terms of plasma glucose, cholesterol, and blood pressure (Sahebkar et al., 2015). The main limitation to the therapeutic use of Resveratrol is its low bioavailability (Alcaín and Villalba, 2009), so novel formulations are currently included in commercially available nutraceuticals, intended for the treatment and prevention of cardio-metabolic disorders (Timmers et al., 2011). In particular, the administration of commercially available nutraceuticals was associated with an improvement of flow-mediated vasodilation, a surrogate measure of endothelial function, in humans (Fujitaka et al., 2011; Wong et al., 2011).

Similar mechanisms, and similar effects on ED and cardiovascular risk, have been proposed also for other flavonoids, such as Quercetin and Fisetin, although fewer studies have been performed with these agents (Gupta et al., 2018; Patel et al., 2018). Similar to Resveratrol, these compounds are frequently included in commercially available nutraceuticals with claimed anti-oxidant properties.

More recently, non-flavonoid small molecules, able to activate SIRT1 were identified and proposed as potential treatments for humans. These compounds can stimulate SIRT1 activity *in vitro* of with a hundred-fold higher potency than Resveratrol (Villalba and Alcaín, 2012). *In vitro* and *in vivo* experiments with SRT1720, a small molecule activator of SIRT1, demonstrated its efficacy in improving mitochondrial biogenesis (Funk et al., 2010), promoting antioxidants expression and blunting inflammation through normalization of NF-κB (Gano et al., 2014). Evidence of the efficacy of this compound in preventing or reducing ED is still limited (Gano et al., 2014; Fiorentino et al., 2015) and no trial in humans has been performed so far. More recently, Scisciola et al. identified two novel potent inhibitors of SIRT1, SCIC2 and its derivative SCIC2.1, which were demonstrated to reduce the progression of cellular senescence *in vitro* (Scisciola et al., 2020). As reported by Charles et al. the actual efficacy of SIRT1 activators is still debatable, due to limitations of the models and validation of the outcomes (Charles et al., 2017).

## Future Perspectives for Basic and Translational Research

Although the role of SIRT1 in aging, and especially in cardiovascular aging, has been extensively studied in the last years, our revision of the literature discloses that still some relevant issues need to be clarified.

From a strictly molecular point of view, the role of SIRT1 in telomeres shortening and nuclear stability, through the interaction with the telomerase complex and lamin A/C, are still poorly understood and need further studies. Similarly, the role of SIRT1 in proteostasis needs to be further investigated. Indeed, the role of SIRT1 and mTOR in autophagy is still largely unclear, whereas a potential role of SIRT1 on the ubiquitin-proteasome system has not been investigated so far.

From a translational point of view, ASCVD seems to associate to a reduction of SIRT1 in tissues, and a paradoxical increase in plasma. This aspect needs to be clarified in details, disclosing the molecular mechanisms underneath. Similarly, the role of SIRT1 gene polymorphisms in cardiovascular risk should be better characterized, in order to investigate whether they are associated with a quantitative or qualitative modification of the protein.

Finally, high-quality clinical trials are necessary to test the effects of PA on SIRT1 and the efficacy of small molecule activators of SIRT1 in reducing cardiovascular risk.

## Conclusions

Sirtuins regulate multiple effector targets involved in multiple cell functions such as mitochondrial respiration, redox balance, apoptosis, cell signaling, and inflammation. All these properties contribute to determine the well-known effect of SIRT1 in preventing cell senescence and prolonging lifespan in animals. Since the cardiovascular system is severely affected by aging, and ASCVD are the most common age-related diseases in industrialized countries, the pathophysiological role of SIRT1 in ASCVD was intensively investigated in the past 20 years. The results of these studies clearly depict a protective role of SIRT1 toward endothelial integrity and function mainly mediated by the increased bio-availability of NO.

Accordingly, the possibility to enhance the activity of SIRT1 through pharmacologic and non-pharmacologic interventions is a promising perspective in the prevention and treatment of ED and ASVCD, although convincing evidence in humans, and particularly in patients, is still missing. Clinical and pre-clinical studies on this promising topic are still ongoing. Accumulating evidence supports the potential role of CR and PA as non-pharmacologic interventions, whereas flavonoids are currently included in nutraceutical products. Pre-clinical studies with small molecule activators of SIRT1 are showing promising data.

## Author Contributions

SM, YP, and LL wrote the initial draft of the manuscript. GB created the pictures. FM and GC contributed to revision and manuscript finalization. All authors contributed to manuscript revision, read, and approved the submitted version.

## Funding

The present work was supported by the Swiss National Science Foundation to GC [310030_197510], and the Foundation for Cardiovascular Research–Zurich Heart House. GC was the recipient of a Sheikh Khalifa's Foundation Assistant Professorship at the Faculty of Medicine, University of Zurich.

## Conflict of Interest

GC is coinventor on the International Patent WO/2020/226993 filed in April 2020. The patent relates to the use of antibodies which specifically bind IL-1α to reduce various sequelae of ischemia-reperfusion injury to the central nervous system. GC is a consultant to Sovida solutions limited. The remaining authors declare that the research was conducted in the absence of any commercial or financial relationships that could be construed as a potential conflict of interest.

## Publisher's Note

All claims expressed in this article are solely those of the authors and do not necessarily represent those of their affiliated organizations, or those of the publisher, the editors and the reviewers. Any product that may be evaluated in this article, or claim that may be made by its manufacturer, is not guaranteed or endorsed by the publisher.
